# Identifying themes of ethical challenges in healthcare leadership during and after the COVID-19 pandemic: a scoping review

**DOI:** 10.1108/LHS-12-2025-0193

**Published:** 2026-07-07

**Authors:** Meri Heiskanen, Eeva Vuorivirta-Vuoti, Minna Ylisirniö, Outi Kanste

**Affiliations:** Research Unit of Health Sciences and Technology, University of Oulu, Oulu, Finland; Research Unit of Health Sciences and Technology, University of Oulu, Oulu, Finland, and Finnish Centre for Evidence-Based Health Care, Helsinki, Finland; Research Unit of Health Sciences and Technology, University of Oulu, Oulu, Finland; Finnish Centre for Evidence-Based Health Care, Helsinki, Finland, and Medical Research Center Oulu, Oulu, Finland

**Keywords:** Leadership, Healthcare, Ethical challenge, COVID-19 pandemic, Scoping review

## Abstract

**Purpose:**

This study aims to describe the themes of ethical challenges identified in healthcare leadership during and after the COVID-19 pandemic.

**Design/methodology/approach:**

This review followed the Joanna Briggs Institute scoping review methodology. A systematic search was done in CINAHL, Scopus, Web of Science, ProQuest, Mednar, PubMed and the Finnish database Medic. The search was conducted from 2020 to 2025, as the scoping review focused on the period during and after the COVID-19 pandemic. Data was analysed using narrative synthesis. The review was reported using the Preferred Reporting Items for Systematic Reviews and Meta-analyses extension for scoping reviews.

**Findings:**

A total of 21 articles were included in the review: 11 research articles and 10 anecdotal articles. Five main themes of ethical challenges identified in healthcare leadership during and after the COVID-19 pandemic were: operating in a turbulent and unpredictable environment; digitalisation and the ethical use of technology; ethical considerations in leadership practice; staff competence and ethical awareness; and patient vulnerability and justice in care.

**Practical implications:**

The findings suggest that organisations need to invest in leadership support, fair policies, workforce sustainability and ethical frameworks that evolve in tandem with technological advancements. Healthcare systems need to strive for greater clarity, purpose and alignment in their data-driven practices. There is a need for ethical training, mentoring and a culture of ethical reflection.

**Originality/value:**

This review produced new, comprehensive information and valuable insights into the existing literature describing ethical challenges in healthcare leadership during and after the COVID-19 pandemic.

## Introduction

Increasing demand for healthcare is driven by population ageing – marked by a higher proportion of older adults with complex care needs – alongside new treatments and rising expectations, particularly in high-income countries. Combined with workforce and funding constraints, these pressures highlight the importance of ethical leadership (Bryden, 2024). Leadership involves influence and typically occurs within groups that share common goals (Northouse, 2022). Based on the recent scientific research, the interest in ethical leadership has continued to grow (Seere *et al.*, 2026; Singh and Vashist, 2024). Ethics refers to the values that an individual or society deems desirable or appropriate. Ethical theory helps leaders to make decisions about what is right or wrong. (Northouse, 2022). The concept of ethics lacks a universally accepted definition and is frequently used interchangeably with morality (Igoumenidis *et al.*, 2026). Similarly, the concept of an ethical challenge remains ambiguously defined and is often conceptualised through related terms such as moral dilemma, ethical conflict and ethical issues (Schofield *et al.*, 2021).

In this review, ethical challenges are defined as situations in which healthcare leaders face uncertainty or conflict regarding the key elements of ethical leadership, such as values, obligations or moral principles (Seere *et al.*, 2026) during and after the COVID-19 pandemic, capturing the uncertainty (Browne and Tie, 2024) experienced by leaders and healthcare personnel alike. Healthcare leaders are broadly defined as individuals in leadership or managerial roles across organisational levels, including senior executives, middle managers, clinical leaders and quality managers. Leadership is thus determined not by profession but by responsibility for guiding, implementing and sustaining quality management processes (Friman *et al.*, 2025).

The global COVID-19 pandemic, which began in 2020 (WHO), complicated the provision and management of healthcare worldwide (Tort-Nasarre *et al.*, 2021; Browne and Tie, 2024) and exposed issues related to the equitable distribution of healthcare resources, including fair access to care (O’Sullivan *et al.*, 2022). It challenged societies worldwide, demonstrating how leadership operates in crises and highlighting the need to redefine leadership to meet the complexities of the modern world (Northouse, 2022; Miklian and Katsos, 2025). COVID-19 also highlighted how healthcare leaders respond in crises and offered lessons on effective leadership (Cariaso-Sugay *et al.*, 2021; Stoller, 2020; Chuku *et al.*, 2025; Mosadeghrad *et al.*, 2024). According to Hofmeyer and Taylor (2021) and Sanabria-Delgado *et al.* (2025), nurse leaders faced severe challenges managing exhausted, fearful staff and making rapid decisions under high organisational demands, while simultaneously battling their own intense emotional fatigue.

COVID-19 also radically accelerated the digital transformation of healthcare, including the rapid adoption of telemedicine and AI-driven tools, which created both opportunities and new ethical concerns related to privacy, fairness and accountability (Robbins *et al.*, 2020; Dulaimy *et al.*, 2024; Borowska Pietrzak *et al.*, 2023; Murphy *et al.*, 2021). Healthcare leaders faced simultaneous challenges, including organising resources for large number of respiratory patients, protecting staff, reorganising services and making ethically complex decisions under extreme pressure (Robbins *et al.*, 2020; Chuku *et al.*, 2025). Moreover, surgical decision-making often remained centred on surgeons’ priorities rather than incorporating nurses’ expertise, thereby limiting nurses’ influence on policies and workflows during the pandemic (Dunn, 2025).

Previous studies have found that during the COVID-19 pandemic, healthcare leaders encountered profound staffing and resource shortages, excessive workloads and significant moral distress, exposing underlying systemic deficiencies in crisis preparedness while underscoring the necessity of resilience and adaptive leadership (Ahlqvist *et al.*, 2023; Tukisi *et al.*, 2025). Leaders had to manage uncertainty about the disease, rapidly changing protocols and shortages of personal protective equipment (PPE) and reorganise services, while adopting flexible and innovative leadership styles, quickly building new processes, supporting staff well-being and ensuring safety (Freitas *et al.*, 2021; Browne and Tie, 2024; Sanabria-Delgado *et al.*, 2025). The nurse leaders were also concerned about the safety of nurses caring for patients with the virus, as they lacked adequate protection against infection. (Tukisi *et al.*, 2025).

Leaders face complex ethical dilemmas, such as balancing financial pressures with doing what is morally right; navigating the ethical implications of digital technologies; and addressing concerns about data privacy, cybersecurity and the ethical use of artificial intelligence (AI) across different organisations and sectors (Ciulla, 2018; Bhatta, 2021). While AI can enhance efficiency, decision-making and transparency, it also raises challenges related to algorithmic bias, fairness and governance, both in healthcare and beyond (Murphy *et al.*, 2021; Kandasamy, 2024). To use AI responsibly, strong ethical principles and oversight are essential, especially in organisations, where leaders must balance both the opportunities and risks AI brings (Brendel *et al.*, 2021).

Healthcare leaders must also reconcile business efficiency with ethical commitments to patient care, ensuring that financial imperatives do not override professional values (Patne and Kanyal, 2024). Many individuals report feelings of isolation and insecurity when making decisions, underscoring the need for support and supervision in navigating complex ethical situations (Skarstein *et al.*, 2024). Healthcare leaders face ethical dilemmas at multiple system levels, including tensions in organisational resource allocation and care quality, the impact of policy and governance on service delivery and broader societal issues of equity and access (Žydžiūnaitė *et al.*, 2010; Ilori *et al.*, 2024).

Despite growing concerns about ethical issues, a preliminary search of Scopus, CINAHL, PROSPERO and the Cochrane Database of Systematic Reviews revealed no recent or ongoing reviews addressing ethical challenges in healthcare leadership. There is a previous review of ethical challenges faced by leaders in technology-driven environments that leverage digital solutions in the digital era (Bhatta, 2021), as well as a review of ethical dilemmas concerning decision-making within healthcare leadership from 2010 (Žydžiūnaitė *et al.*, 2010). However, none provides a comprehensive overview of the ethical challenges faced in healthcare leadership during and after the COVID-19 pandemic. Thus, this study aimed to describe themes of ethical challenges identified in healthcare leadership during and after the COVID-19 pandemic.

The review question was as follows:

*RQ1*. What kinds of themes of ethical challenges have been identified in healthcare leadership during and after the COVID-19 pandemic?

## Research methods

### Study design

A scoping review was conducted in accordance with the Joanna Briggs Institute scoping review methodology. A scoping review was conducted because information about ethical challenges in healthcare leadership is scattered, and a comprehensive overview is needed (Peters *et al.*, 2020). The review was reported according to the Preferred Reporting Items for Systematic Reviews and Meta-analyses extension for scoping reviews (PRISMA-Scr) (Page *et al.*, 2021; Supplementary File 1), and the review was registered on the Open Science Framework (blinded for review).

### Inclusion and exclusion criteria

The inclusion and exclusion criteria were defined using the PCC framework (participants, concept and context) (Peters *et al.*, 2020; see Table 1). Inclusion criteria for participants were that they were healthcare leaders. The time frame was limited from January 2020 to August 2025, as the scoping review focused on the period during and after the COVID-19 pandemic.

**Table 1.** Inclusion and exclusion criteria

PCC	Inclusion criteria	Exclusion criteria
Participants (P)	Healthcare leaders (e.g. nurse leaders, healthcare leaders, supervisors, administrators, managers)	Other healthcare professionals, students and patients
Concept (C)	Ethical challenges in healthcare leadership	Not related to ethical challenges in healthcare leadership
Context (C)	Healthcare organisations and the healthcare sector in any geographic location	Not related to the healthcare sector or organisations
Type of source	Studies using quantitative, qualitative and mixed methods designs, as well as unpublished studies, theoretical papers, features, case study scenarios and journal articles. Published in English, Finnish or Swedish. Studies from January 2020 to August 2025	Books, thesis, reviews

Note(s):

If a study includes professionals other than leaders, the minimum requirement is that leaders constitute at least 50% of the participants

**Source(s):** Authors’ own work

### Data sources and search strategy

A three-step search strategy was used to locate both published and unpublished studies. The first step involved an initial limited search of Scopus and CINAHL. The words contained in the titles and abstracts of relevant articles, as well as the index terms used to describe them, were used to develop a comprehensive strategy (Supplementary File 2). In the second search step, the identified keywords and index terms were used to search CINAHL, Scopus, Web of Science, ProQuest, Mednar, PubMed and the Finnish database Medic. The search strategy was developed in consultation with an information specialist. Searches were conducted in August 2025. Studies published in English, Finnish or Swedish were included in this review due to the challenges associated with acquiring and translating studies in other languages. In the third step, reference lists of all included articles were searched for additional relevant studies.

### Study selection and data extraction

**Figure 1.**
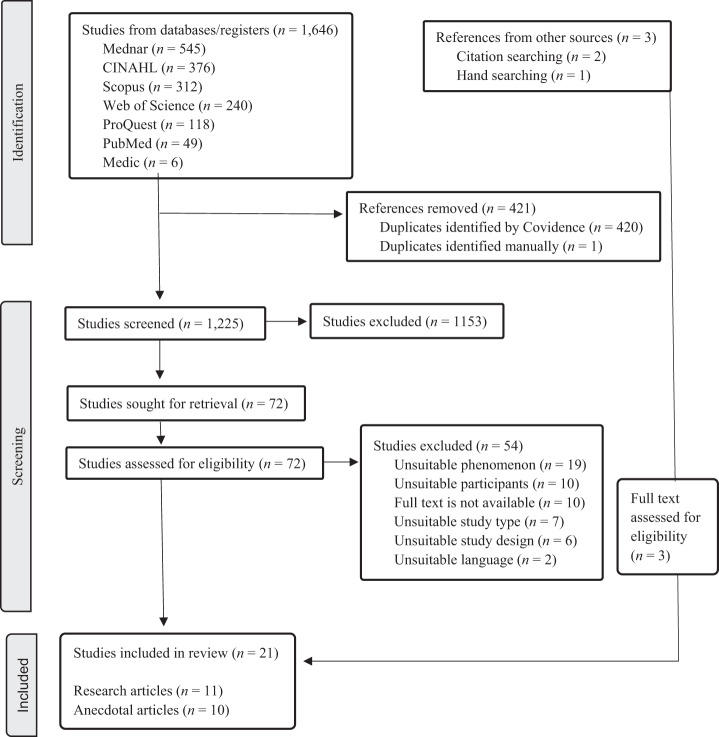
Illustration of the search process and study selection in the PRISMA flow diagram **Source:** Authors’ own work

All the records identified (*n* = 1,646) in preliminary, manual and citation searches were collated and uploaded to the Covidence bibliographic management system (Covidence Systematic Review Software, 2023). Duplicates (*n* = 421) were then removed, leaving 1,225 articles. Screening of the titles and abstracts of these articles by two independent authors (blinded for review) using the PCC framework resulted in the exclusion of 72 articles. After reviewing the full texts of the remaining 72 articles, 18 were selected for inclusion. A list of the excluded full-text studies is provided in Supplementary file 3. Three articles were identified through citation searching of the included studies (*n* = 2) and manual searching in Scopus (*n* = 1). The article selection process is presented in the PRISMA flow diagram (Figure 1). Any disagreements between the reviewers (blinded for review) were resolved through mutual discussion. The data to be extracted included information on the authors, publication year, publication type, country of origin, study aims, methodology and key findings.

### Data analysis and presentation of results

**Figure 2.**
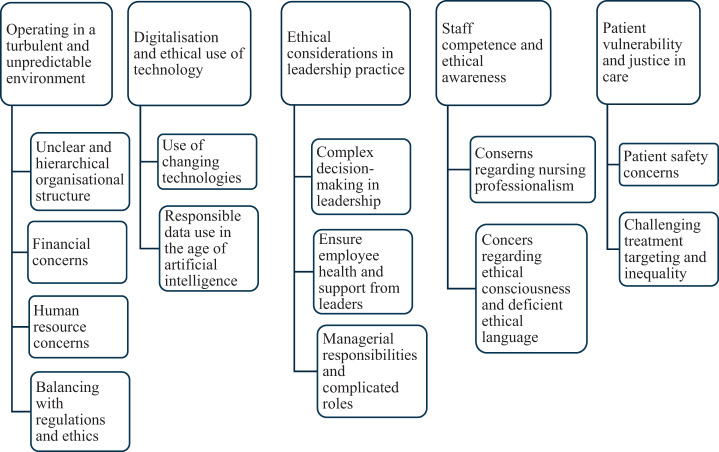
Themes identifying ethical challenges in healthcare leadership during and after the COVID-19 pandemic **Source:** Authors’ own work

The extracted data were systematically analysed using a narrative synthesis approach. The key findings of the articles were carefully reviewed, and the emerging themes were grouped into sub-themes and subsequently into main themes based on their similarity (Peters *et al.*, 2020; see Figure 2). The data is presented in tabular and graphical formats, accompanied by a narrative summary.

## Results

### Characteristics of included articles

The review encompassed 21 articles, including 11 research articles and 10 anecdotal articles. Eight of research articles originated from the USA, while the rest came from Finland (*n* = 2), Norway (*n* = 2), Brazil (*n* = 1), Indonesia (*n* = 1), Sweden (*n* = 1), the UK (*n* = 1), Nepal (*n* = 1), Italy (*n* = 1) and Ireland (*n* = 1). Two articles have many origin countries (Germany, Austria, Switzerland, the USA, Germany and the USA). Of the 11 research articles, 9 used a qualitative design and 2 used a quantitative design. The number of participants ranged from 10 to 316, for a total of 759 individuals (Supplementary Files 4 and 5).

### Review findings

Five main themes describing ethical challenges in healthcare leadership during and after the COVID-19 pandemic were identified:

1. operating in a turbulent and unpredictable environment;
2. digitalisation and ethical use of technology;
3. ethical considerations in leadership practice;
4. staff competence and ethical awareness; and
5. patient vulnerability and justice in care (Figure 2).

### Operating in a turbulent and unpredictable environment

The first main theme included sub-themes of unclear and hierarchical organisational structure, financial concerns, human resource concerns and balancing with regulations and ethics.

*Unclear and hierarchical organisational structure* encompassed a lack of individual and organisational integrity (Beil-Hildebrand *et al.*, 2024). Nurse leaders at ward, middle and strategic levels experience insufficient support from organisational administration. They feel that, in the ward, operations are planned on doctors’ terms without taking nurses’ input into account (Aitamaa *et al.*, 2021). Nurses feel undervalued, and their expertise is often overlooked or disrespected (Beil-Hildebrand *et al.*, 2024).

*Financial concerns* included the negative impact of finances on care (Beil-Hildebrand *et al.*, 2024), the quality of nursing, the sufficiency of financial resources for development work and concerns about downsizing personnel due to cost-cutting measures (Aitamaa *et al.*, 2021). Nurse leaders need to ensure high-quality care, foster inclusive stakeholder engagement and maintain a healthy, supportive environment, all while addressing organisational and financial priorities (Rushton *et al.*, 2025). In addition, senior management, comprising nurse leaders and the unit head, appeared to prioritise budgetary constraints over fostering an ethical climate (Storaker *et al.*, 2022).

Human resource concerns encompassed challenges in resource allocation during the COVID-19 pandemic (Aitamaa *et al.*, 2021; Lesandrini and Reis, 2022), maintaining high standards despite poor resources (Arjama *et al.*, 2025; Ghimire and Neupane, 2025), managing limited resources (Rushton, 2025), workforce shortages (Stucky and Wymer, 2023) and sufficiency of human resources for development work (Aitamaa *et al.*, 2021). The shortage of nurses presents an ethical challenge for nurse leaders (Downs *et al.*, 2022). Nurse leaders also felt bad when they promised family members that they would provide individual care for a resident, knowing that it would not be possible due to staffing shortages (Arjama *et al.*, 2025).

*Balancing with regulations and ethics* included challenges when leaders need to be able to make ethical decisions even when instructions are incomplete or subject to rapid change (Arjama *et al.*, 2025). In establishing guidelines and policies for ethical conduct, healthcare leaders need to apply the principles of professionalism to new situations. This is particularly important when organisations need to provide practical strategies that maximise the benefits of social media while also respecting ethical considerations. Employees should also be educated and supported in the appropriate use of social media. (Ennis-O-Connor and Mannion, 2020). Balancing regulations with daily care needs is also challenging. This is because statistics and monitoring take time away from nurse leaders’ working hours. However, this does not promote the well-being of residents or staff. Furthermore, nurse leaders were unaware of who benefited from the information. (Arjama *et al.*, 2025).

### Digitalisation and ethical use of technology

The second main theme included sub-themes of the use of changing technologies and responsible data use in the age of AI.

The use of *changing technologies* highlights the ethical challenges nurse leaders face in an increasingly digital healthcare environment (Stucky and Wymer, 2023; Sullivan *et al.*, 2024). They struggle with decisions regarding data accessibility, admit to having privacy concerns and the need to remain current with emerging tools to manage resources effectively (Sullivan *et al.*, 2024). These challenges were acute during the COVID-19 pandemic, when nurse leaders had to make critical decisions based on incomplete data while adapting to shifting operational conditions and navigating conflicting organisational and governmental directives (Stucky and Wymer, 2023). As new staffing models such as telehealth continue to expand, bringing clear benefits to patients, communities and the wider healthcare system. However, leaders must also carefully consider the ethical implications these innovations create for all stakeholders involved (Lesandrini and Reis, 2022).

*Responsible data use in the age of AI* raises profound ethical questions about integrating AI into healthcare. Nurse leaders need to ensure that digital processes are translated into understandable, ethically sound and clinically relevant information. They also need to ensure that AI applications promote equality, inclusion and justice rather than perpetuate disparities. (Arcadi, 2025). Furthermore, leaders need to address nurses’ concerns about the potential erosion of human interaction and to foster trust in the utilisation of AI-generated data (Sullivan *et al.*, 2024). They must uphold and cultivate an organisational culture in which person-to-person relationships remain central. By making time for personal interaction and encouraging emotionally intelligent leadership, nurses can foster collaborative environments that promote the well-being of patients and healthcare teams alike. (Arcadi, 2025).

### Ethical considerations in leadership practice

The third main theme included sub-themes of complex decision-making in leadership, ensure employee health and support from leaders and managerial responsibilities and complicated roles.

*Complex decision-making in leadership* encompassed the fact that leaders are standing alone in decision-making during the COVID-19 pandemic, and they are forced to make decisions based on uncertainty rather than evidence (Stucky and Wymer, 2023; Karlsson *et al.*, 2024). They need to maintain their ability to make ethical decisions when instructions are incomplete or rapidly changing (Arjama *et al.*, 2025). Senior nursing leaders are often responsible for balancing multiple competing priorities and considerations when making decisions about potential workforce strategies and care models (Rushton *et al.*, 2025). Nurse leaders often report feeling isolated when confronted with ethical challenges (Arjama *et al.*, 2025). In navigating such dilemmas, it is essential for them to critically evaluate which values are being upheld or compromised by each potential decision.

*Ensure employee health and support from leaders* included that leaders often struggle to balance the demands of supporting their staff’s occupational well-being while also attending to their own (Arjama *et al.*, 2025). In the digital age, their role has expanded to include helping employees navigate and uphold ethical responsibilities in increasingly complex work environments (Ennis-O-Connor and Mannion, 2020). Concerns in hospitals have also been raised about perceived unfairness in how crisis pay was distributed during the COVID-19 pandemic (Chipps *et al.*, 2022) and about challenges related to nurses’ experiences of being heard and receiving positive feedback (Aitamaa *et al.*, 2021). Importantly, staff well-being is closely linked to the quality of care they provide (Downs *et al.*, 2022). Leaders in residential care facilities need to navigate the difficult task of protecting staff safety while managing the inherent risks of working in potentially infectious environments during the COVID-19 pandemic (Karlsson *et al.*, 2024), including in tertiary care centres, ensuring the safety of resuscitation teams during the COVID-19 pandemic in situations where PPE is required (Lahey *et al.*, 2021).

*Managerial responsibilities and complicated roles* encompassed the fact that nurse leaders feel that they do not have enough time to fulfil all their responsibilities as leaders. They also found it difficult to take days off, and if they did, the backlog of tasks further increased their sense of burden. (Arjama *et al.*, 2025). Nurse leaders face challenges in fully embodying their leadership roles and have expressed concerns about ensuring that staff feel comfortable reaching out to them at any time (Devik *et al.*, 2020). They also have an unreasonable scope of responsibility and may be held accountable for patient and organisational outcomes (Rushton *et al.*, 2025). They often face competing demands, ranging from participating in budget meetings to addressing complex patient situations (Storaker *et al.*, 2022). Common challenges include a lack of appropriate tools, limited time and inconsistent motivation among team members (Devik *et al.*, 2020). Leaders’ lack of clarity about their core values can lead to confusion and uncertainty among staff and teams (Rushton *et al.*, 2025).

Despite these difficulties, they are expected to model ethical behaviour and effective leadership, setting a standard for their colleagues and acting as role models for junior staff (Lesandrini and Leclerc, 2024; Nopita Wati *et al.*, 2023). Support from supervisors or senior managers affected how well nurse leaders could act ethically (Arjama *et al.*, 2025). In the post-pandemic era, leaders have become increasingly vocal in addressing injustice and inequitable practices within healthcare settings (Musbahi *et al.*, 2022).

### Staff competence and ethical awareness

The fourth main theme included sub-themes of concerns regarding nursing professionalism and concerns regarding ethical consciousness and deficient ethical language.

Concerns regarding nursing professionalism encompassed, for instance, incompetence, misbehaviour, dishonesty and poor teamwork (Beil-Hildebrand *et al.*, 2024), and in hospitals showing work despite feeling unwell or not performing at full capacity (Cunha *et al.*, 2023). The departure of skilled nurses adversely affects workload and workforce sustainability. While it signifies career advancement for the individual, it is also a loss for the healthcare team. (Ghimire and Neupane, 2025) There are also ethical concerns about the increasing demand for nurses to have a high level of competence. For example, Storaker *et al.* (2022) found that procedures that were once performed by physicians are now performed by nurses.

*Concerns regarding ethical consciousness and deficient ethical language* included leaders’ concerns about the lack of ethical terms or language. Although ethical issues were evident in some situations, they were not addressed. Nurse leaders were concerned about young nurses; they seemed immature and less reflective than in previous years. They no longer subscribe to the idea of nursing as a calling. (Storaker *et al.*, 2022) To recognise ethically challenging situations, leaders are present when staff are providing daily care (Arjama *et al.*, 2025).

### Patient vulnerability and justice in care

The fifth main theme included sub-themes of patient safety concerns and challenging treatment targeting and inequality.

*Patient safety concerns* included issues impacting patient safety and healthcare quality (Stucky and Wymer, 2023). Patient safety concerns can include problems with skills, excessive workload, staff shortages, lack of support services, time constraints that prevent nursing care, lack of access to specialist care and lack of palliative care and effective pain management (Beil-Hildebrand *et al.*, 2024). In addition, the departure of skilled nurses adversely affects patient safety (Ghimire and Neupane, 2025). Nurse leaders also knew that the safety of staff and residents could not always be guaranteed, which worked against them (Arjama *et al.*, 2025). However, nurse leaders are responsible for ensuring patient safety (Rushton *et al.*, 2025). Having to restrict visits during the COVID-19 pandemic, although aware of residents’ need for personal connection and protecting the group and promoting the autonomy of older people, presented an ethical challenge (Chipps *et al.*, 2022; Lahey *et al.*, 2021; Lesandrini and Reis, 2022; Karlsson *et al.*, 2024).

*Challenging treatment targeting and inequality* encompassed nurse leaders’ understanding how some groups may be privileged (Musbahi *et al.*, 2022) and fair ventilation allocation during the COVID-19 pandemic (Lahey *et al.*, 2021). The use of technologies such as facial recognition can also lead to unequal treatment and reduce access to appropriate care. Studies have shown that algorithms used to predict healthcare needs often underestimate the severity of illness in black patients compared to white patients with the same algorithmic risk score, and that the error rate is higher when identifying people of colour compared to white individuals. (Arcadi, 2025). The loss of skilled workers exacerbated existing inequalities and undermined the fundamental right to healthcare (Ghimire and Neupane, 2025).

## Discussion

This review produced new, comprehensive information and valuable insights into the existing literature describing ethical challenges in healthcare leadership during and after the COVID-19 pandemic. Five main themes were identified: operating in a turbulent and unpredictable environment, digitalisation and ethical use of technology, ethical considerations in leadership practice, staff competence and ethical awareness and patient vulnerability and justice in care. This review also highlights context-specific ethical tensions and emerging leadership demands that have not been systematically synthesised previously.

The results of our review highlighted a lack of individual and organisational integrity, and that planning operations are often conducted on doctors’ terms without considering nurses’ input, providing new insights. Our findings align with previous studies (Dunn, 2025), which prove that surgical planning and decision-making in perioperative care are typically organised around surgeons’ needs and schedules, rather than nurses’ input. Nurses possess critical expertise but have limited authority to influence workflow, policies or scheduling.

Our findings also revealed ethical challenges, including financial and human resource concerns. Nurse leaders need to ensure high-quality care, maintain a healthy, supportive environment, all while addressing organisational and financial priorities. Our results support other studies highlighting resource concerns, in which leadership is challenged by a lack of workforce and funding (Bryden, 2024). In addition, leaders face dilemmas such as balancing financial pressures with what is morally right (Ciulla, 2018; Seere *et al.*, 2026). Our results also align with the results of Patne and Kanyal (2024), who identified that hospital management faces the challenge of ensuring that financial goals do not override core healthcare values, and with Žydžiūnaitė *et al.* (2010) and Ilori *et al.* (2024), who discovered that healthcare leaders face concerns at the institutional level, such as balancing resource allocation and maintaining care quality.

The findings of this review revealed ethical challenges in balancing regulatory tasks with daily care that have not been clearly noted previously, providing new and valuable insights into the existing literature. Collecting and monitoring data took time away from nurse leaders without clearly benefiting residents or staff. To overcome this, healthcare leaders should apply professional principles to changing situations, particularly when developing ethical strategies that effectively utilise tools such as social media. Patne and Kanyal (2024) have identified that hospital management faces complex ethical implications when attempting to balance profitability with patient-centred ethics.

Our results reveal significant ethical challenges related to data privacy, accessibility and the use of AI in healthcare. These concerns align with previous research (Bhatta, 2021; Robbins *et al.*, 2020; Dulaimy *et al.*, 2024; Borowska Pietrzak *et al.*, 2023; Murphy *et al.*, 2021). Our findings indicate that the rapid adoption of telemedicine and AI-driven tools has created both opportunities and new ethical concerns related to privacy, fairness and accountability. These systems need to promote equality, inclusion and justice, rather than reinforcing existing disparities. Our results align with those of Kandasamy (2024), who cautions that biased algorithms can yield unfair outcomes that disadvantage certain groups.

Our results indicate that leaders must address healthcare professionals’ concerns, particularly about the potential erosion of human interaction, and strive to build trust in the use of AI-generated data. At the same time, the COVID-19 pandemic forced healthcare systems to rapidly adapt to managing high patient volumes, protecting staff and safeguarding vulnerable populations (Robbins *et al.*, 2020; Chuku *et al.*, 2025). This period of accelerated digitalisation introduced both opportunities and new ethical challenges for leadership in healthcare and beyond (Borowska Pietrzak *et al.*, 2023). While the adoption of AI in healthcare appears inevitable, ethical and regulatory frameworks have not kept pace with its rapid development (Murphy *et al.*, 2021). To ensure the responsible use of AI, organisations need to adopt clear ethical principles (Brendel *et al.*, 2021).

The findings indicate that during the COVID-19 pandemic, leaders were often left to make decisions alone, feeling a lack of support from organisational administration and were forced to make decisions based on uncertainty rather than evidence. This finding aligns with Skarstein *et al.* (2024), who found that healthcare leaders often feel lonely in their leadership positions, and Ahlqvist *et al.* (2023), who discovered that nurse leaders experience responsible overload. These circumstances contribute to the difficulty of making morally sound decisions in ethically charged environments (Ciulla, 2018). Our results also emphasise that support from senior leadership is crucial to enabling nurse leaders to act ethically. The presence or absence of such support directly affects their capacity to navigate ethical dilemmas effectively. This aligns with the results of Skarstein *et al.* (2024) and Ahlqvist *et al.* (2023), who found that healthcare and nursing leaders need emotional support and supervision.

Our results showed that nurse leaders face ethical challenges in balancing support for their staff and their own well-being, as well as the unfairness in how crisis pay was distributed during the pandemic. Nurse leaders have concerns about ensuring that staff feel comfortable reaching them at any time. Our results support other studies (Hofmeyer and Taylor, 2021; Sanabria-Delgado *et al.*, 2025; Ahlqvist *et al.*, 2023; Browne and Tie, 2024; Chuku *et al.*, 2025) that showed nurse leaders face the dual pressures of supporting exhausted, fearful staff while making rapid decisions in constantly changing conditions. In addition, leaders were required to implement new strategies in practice to support the resilience of nursing staff, for example, regarding psychological safety (Mosadeghrad *et al.*, 2024). Many reported high stress, moral distress and responsibility overload as they managed crisis communication, staffing shortages and emotional support needs.

Our results showed that nurse leaders do not have enough time to fulfil their responsibilities and find it difficult to take days off. Despite these challenges, they are expected to model ethical behaviour, set a standard for their colleagues and act as role models for junior staff. Also, Ahlqvist *et al.* (2023) noted that nurse leaders reported responsibility overload. In the post-pandemic era, leaders have become increasingly vocal in addressing injustice and inequitable practices within healthcare settings.

Our review highlights ethical challenges, including a lack of nursing professionalism, ethical consciousness and deficient ethical language. Reports included cases of insufficient competence, unprofessional conduct, lack of honesty, poor collaboration and instances in which staff continued working despite being unwell. These issues are further compounded by the departure of experienced nurses, which adversely affects workload and workforce sustainability. Žydžiūnaitė *et al.* (2010) also noted that leaders in healthcare face concerns related to ethical tensions that professionals’ expertise, and furthermore, Ilori *et al.* (2024) noted that leaders must ensure professionals’ ethical integrity. Our findings were the first to reveal that nurse leaders expressed concern over the absence of ethical terminology in staff discussions, even when clear ethical issues were present. In addition, nurse leaders were concerned about young nurses; they appeared less mature and less reflective than in previous years.

Our findings were the first to highlight ethical challenges related to patient safety, providing valuable insights. Patient safety was adversely affected by inadequate competencies, limited support services, restricted access to specialised and palliative care, shortcomings in effective pain management, the loss of experienced nurses, excessive workloads, staff shortages and time pressures, all of which hindered the provision of proper nursing care. Pandemic-related visitation restrictions added to these challenges. Nurse leaders also knew that the safety of staff and residents could not always be guaranteed, however they are responsible for ensuring that. Similarly, in other studies (Tukisi *et al.*, 2025; Sanabria-Delgado *et al.*, 2025), nurse leaders reported critical shortages of human and material resources, high overtime, the addition of many new wards and patients and reduced staffing and equipment during the COVID-19 pandemic. The nurse leaders were concerned about the safety of nurses caring for patients with the virus, as they lacked adequate protection against infection.

The results provide valuable insights from leaders concerning the equitable distribution of care, including the fair allocation of ventilators and the privilege afforded to some groups during the COVID-19 pandemic. In addition, leaders are concerned about the ethical implications of using facial recognition technologies, which may lead to inequalities in healthcare. These findings reinforce the view that ethical, evidence-based guidelines are necessary in healthcare decision-making (O’Sullivan *et al.*, 2022; Seere *et al.*, 2026). In addition, there is an urgent need for ethical guidelines for the use of AI-based solutions in healthcare (Brendel *et al.*, 2021). Furthermore, our findings indicate that the loss of skilled healthcare workers during the pandemic has deepened existing inequalities and posed a significant ethical challenge by undermining the fundamental right to healthcare.

### Practical implications

Based on this review, healthcare organisations should strengthen leadership support and develop evolving ethical frameworks to balance financial responsibilities with patient-centred care, addressing concerns such as data privacy, inequality, algorithmic bias, transparency and accountability. Ensuring safe and equitable care requires strengthened support for nurse leaders, investment in workforce sustainability and the development of inclusive, bias-aware digital tools and decision-making models, alongside fair policies and adequate resources to uphold ethical practice and high-quality care.

The review highlights the need to strengthen ethical professionalism in nursing through strong leadership, structured ethics training, mentoring and a culture supporting ethical reflection. Regular training and team-based reflection sustain ethical competence, enhance decision-making and help address ethical dilemmas, thereby improving patient care.

### Limitations

One limitation in conducting this review was that the term “ethical challenge” is difficult to define, making it difficult to select relevant search terms (Cunha *et al.*, 2023; Storaker *et al.*, 2022; Seere *et al.*, 2025). As a result, we may have overlooked some relevant publications. Some relevant studies may also have been excluded due to the limited number of languages in the search strategy. Although the included studies cover diverse geographical contexts, they do not represent all healthcare settings; thus, findings should be interpreted contextually (Peters *et al.*, 2020). Narrative synthesis was conducted by a single researcher, increasing the potential for subjectivity in the analysis (Peters *et al.*, 2020). To mitigate this risk, the analysis was discussed and verified by other researchers to enhance credibility.

## Conclusions

This scoping review identified a wide range of ethical challenges faced by healthcare leaders during and after the COVID-19 pandemic, highlighting their complexity. These challenges often arise from hierarchical structures, resource concerns, the need to balance regulatory requirements, complex decision-making processes, significant responsibilities, a lack of professionalism and ethical awareness, patient safety concerns and inequality.

Nurse leaders face substantial ethical burdens while lacking sufficient time, support and resources to fulfil their roles. Recognising and addressing these dilemmas is essential for ethically sound, person-centred leadership. Healthcare systems must enhance clarity and alignment in data-driven practices, invest in workforce sustainability and develop evolving ethical frameworks addressing data privacy, inequality, algorithmic bias, transparency and accountability. Strengthening ethical professionalism through leadership, training, mentoring and reflective practices is critical, as regular training and team reflection support ethical competence, decision-making and effective management of ethical dilemmas.

Future research should focus on developing actionable ethical leadership frameworks and context-sensitive guidelines that enable healthcare leaders to make ethically sound decisions in complex environments, as well as institutional support systems to facilitate this. Furthermore, studies should examine interventions that enhance ethical competence, promote inclusive and person-centred care.

## Supplementary Material

Data supplement 1

## Data Availability

The data that supports the findings of this study are available in the supplementary material of this article.

## Supplementary material

The supplementary material for this article can be found online.
