# Supplementary material for: Identifying themes of ethical challenges in healthcare leadership during and after the COVID-19 pandemic: a scoping review
Source: Leadersh Health Serv (Bradf Engl). 2026 Jul 7;39(5):116–32. doi: 10.1108/LHS-12-2025-0193 (PMC13340037; doi:10.1108/LHS-12-2025-0193)
Supplement: Data supplement 1 [file lhs-12-2025-0193_supplementary_file.docx]

Supplementary Files 1-5.

Supplementary File 1. PRISMA-ScR Checklist for Scoping Reviews

**Preferred Reporting Items for Systematic reviews and Meta-Analyses extension for Scoping Reviews (PRISMA-ScR) Checklist**

| **SECTION** | **ITEM** | **PRISMA-ScR CHECKLIST ITEM** | **REPORTED ON PAGE #** |
| --- | --- | --- | --- |
| **TITLE** | | | |
| Title | 1 | Identify the report as a scoping review. | Title page |
| **ABSTRACT** | | | |
| Structured summary | 2 | Provide a structured summary that includes (as applicable): background, objectives, eligibility criteria, sources of evidence, charting methods, results, and conclusions that relate to the review questions and objectives. | Abstract page |
| **INTRODUCTION** | | | |
| Rationale | 3 | Describe the rationale for the review in the context of what is already known. Explain why the review questions/objectives lend themselves to a scoping review approach. | 1-3 |
| Objectives | 4 | Provide an explicit statement of the questions and objectives being addressed with reference to their key elements (e.g., population or participants, concepts, and context) or other relevant key elements used to conceptualize the review questions and/or objectives. | 4, Table 1 |
| **METHODS** | | | |
| Protocol and registration | 5 | Indicate whether a review protocol exists; state if and where it can be accessed (e.g., a Web address); and if available, provide registration information, including the registration number. | 3 |
| Eligibility criteria | 6 | Specify characteristics of the sources of evidence used as eligibility criteria (e.g., years considered, language, and publication status), and provide a rationale. | 4 |
| Information sources* | 7 | Describe all information sources in the search (e.g., databases with dates of coverage and contact with authors to identify additional sources), as well as the date the most recent search was executed. | 4 |
| Search | 8 | Present the full electronic search strategy for at least 1 database, including any limits used, such that it could be repeated. | 4, Supplementary File 2 |
| Selection of sources of evidence† | 9 | State the process for selecting sources of evidence (i.e., screening and eligibility) included in the scoping review. | 4-5, Figure 1, Supplementary File 3 |
| Data charting process‡ | 10 | Describe the methods of charting data from the included sources of evidence (e.g., calibrated forms or forms that have been tested by the team before their use, and whether data charting was done independently or in duplicate) and any processes for obtaining and confirming data from investigators. | - |
| Data items | 11 | List and define all variables for which data were sought and any assumptions and simplifications made. | - |
| Critical appraisal of individual sources of evidence§ | 12 | If done, provide a rationale for conducting a critical appraisal of included sources of evidence; describe the methods used and how this information was used in any data synthesis (if appropriate). | - |
| Synthesis of results | 13 | Describe the methods of handling and summarizing the data that were charted. | 5 |
| **RESULTS** | | | |
| Selection of sources of evidence | 14 | Give numbers of sources of evidence screened, assessed for eligibility, and included in the review, with reasons for exclusions at each stage, ideally using a flow diagram. | 4-5 |
| Characteristics of sources of evidence | 15 | For each source of evidence, present characteristics for which data were charted and provide the citations. | Supplementary File 3 and 4 |
| Critical appraisal within sources of evidence | 16 | If done, present data on critical appraisal of included sources of evidence (see item 12). | - |
| Results of individual sources of evidence | 17 | For each included source of evidence, present the relevant data that were charted that relate to the review questions and objectives. | 5-6 |
| Synthesis of results | 18 | Summarize and/or present the charting results as they relate to the review questions and objectives. | 5-6 |
| **DISCUSSION** | | | |
| Summary of evidence | 19 | Summarize the main results (including an overview of concepts, themes, and types of evidence available), link to the review questions and objectives, and consider the relevance to key groups. | 6-11, Figure 2 |
| Limitations | 20 | Discuss the limitations of the scoping review process. | 15 |
| Conclusions | 21 | Provide a general interpretation of the results with respect to the review questions and objectives, as well as potential implications and/or next steps. | 15-16 |
| **FUNDING** | | | |
| Funding | 22 | Describe sources of funding for the included sources of evidence, as well as sources of funding for the scoping review. Describe the role of the funders of the scoping review. | Title page |

JBI = Joanna Briggs Institute; PRISMA-ScR = Preferred Reporting Items for Systematic reviews and Meta-Analyses extension for Scoping Reviews.

* Where *sources of evidence* (see second footnote) are compiled from, such as bibliographic databases, social media platforms, and Web sites.

† A more inclusive/heterogeneous term used to account for the different types of evidence or data sources (e.g., quantitative and/or qualitative research, expert opinion, and policy documents) that may be eligible in a scoping review as opposed to only studies. This is not to be confused with *information sources* (see first footnote).

‡ The frameworks by Arksey and O’Malley (6) and Levac and colleagues (7) and the JBI guidance (4, 5) refer to the process of data extraction in a scoping review as data charting*.*

§ The process of systematically examining research evidence to assess its validity, results, and relevance before using it to inform a decision. This term is used for items 12 and 19 instead of "risk of bias" (which is more applicable to systematic reviews of interventions) to include and acknowledge the various sources of evidence that may be used in a scoping review (e.g., quantitative and/or qualitative research, expert opinion, and policy document).

*From:* Tricco AC, Lillie E, Zarin W, O'Brien KK, Colquhoun H, Levac D, et al. PRISMA Extension for Scoping Reviews (PRISMAScR): Checklist and Explanation. Ann Intern Med. 2018;169:467–473. doi: 10.7326/M18-0850

Supplementary File 2. Database, search strategy and results from databases.

| Database | Search strategy | *n* |
| --- | --- | --- |
| CINAHL | ((MH "Ethics, Professional+" OR MH "Decision Making, Ethical") AND (MH "Leadership") OR (ethic* N2 leader*)) AND ((MH "Health Services Administration+" OR MH "Hospitals+" OR MH "Health Occupations+" OR healthcare OR “health care” OR nursing OR hospital OR hospitals OR “primary care”)) AND EM 20250201- | 376 |
| Scopus | ( TITLE-ABS-KEY ( ethic* W/2 leader* ) AND TITLE-ABS-KEY ( healthcare OR "health care" OR nursing OR hospital OR hospitals OR "primary care" ) ) AND ORIG-LOAD-DATE > 20250201  Hand search was performed in the Scopus database using the search term 'ethical challenges in healthcare leadership´ (n = 1). | 312 |
| Web of Science | ethic* NEAR/2 leader* (Title) and healthcare OR “health care” OR nursing OR hospital OR hospitals OR “primary care” (Topic) ja index date 2025-02-01 to 2025-08-29 | 240 |
| ProQuest | Noft(ethic* NEAR/2 leader*) AND noft(healthcare OR “health care” OR nursing OR hospital OR hospitals OR “primary care”) ja additional limits – date: after 01 February 2025 | 118 |
| MedNar | ethic* AND leader* AND (healthcare OR “health care” OR nursing OR hospital OR hospitals OR “primary care”) ja datelimit 20205-2025 | 545 |
| PubMed | (("Ethics, Professional"[Mesh] AND "Leadership"[Mesh]) OR "ethical leadership"[Title/Abstract:~2] OR "ethics leadership"[Title/Abstract:~2] OR "ethical leader"[Title/Abstract:~2] OR "ethics leader"[Title/Abstract:~2]) AND (("Health Care Category"[Mesh] OR "Health Occupations"[Mesh]) OR (healthcare[Title/Abstract] OR "health care"[Title/Abstract] OR nursing[Title/Abstract] OR hospital[Title/Abstract] OR hospitals[Title/Abstract] OR "primary care"[Title/Abstract])) AND (("2025/02/01"[CRDT] : "2026"[CRDT] OR "2025/02/01"[EDAT] : "2026"[EDAT] )) | 49 |
| The Finnish database Medic | ethic* OR etiik* OR eetti* AND johta* OR leader* ja 2025-2025 | 6 |

Note: Searches were conducted in August 2025, and the time was limited from 2020 to 2025. Source: Authors´ own work.

Supplementary File 3. Excluded full texts and the reason for exclusion.

| Study | Reason for exclusion |
| --- | --- |
| Ahmed, M. and Khan, M.I. (2024). “Unpacking the paradoxical impact of ethical leadership on employees’ pro-social rule breaking behavior: the interplay of employees’ psychological empowerment and moral identity”. *Current Psychology*, Vol 43, pp. 29275–29290. | Unsuitable participants |
| Angelos, P., Devon, K., Ferreres, A. R., McLeod, R., and Ellison, E. C. (2021). “A crucial moment for reflection on the importance of ethical leadership in academic medicine”, *Annals of Surgery,* Vol 273 No. 2, e33–e35. | Unsuitable phenomenon |
| Anawati, A. and Verma, S. (2021). “Commentary: Moral and Ethical Leadership in the Age of Diversity, Equity, Inclusiveness and Social Accountability”, *Healthcare Quarterly, Vol 24 No. 3, pp.* 18–22. | Full text is not available |
| Arjanto, P., Bafadal, I., Atmoko, A., and Sunandar, A. (2020). “From ethical principles to practice: The growing importance of moral leadership in education”, *European Journal of Contemporary Education*, Vol 12 No. 4, pp. 1150–1165. | Unsuitable study design |
| Australian Institute of Health and Safety. (2024). “Navigating the challenges of ethical leadership in OHS: As organisational pressures challenge OHS principles, professionals must navigate ethical dilemmas to uphold worker safety and integrity”, *OHS Professional*. | Full text is not available |
| Ayala-Yáñez, R., Ruíz-López, R., McCullough, L.B. and Chervenak, F.A. (2020). ”Violence against trainees: urgent ethical challenges for medical educators and academic leaders in perinatal medicine”, *Journal of Perinatal Medicine*, Vol 48. No. 7, pp. 728-732. | Unsuitable study design |
| Awad, N. H. A. and Ashour, H. M. A. (2022). “Crisis, ethical leadership and moral courage: Ethical climate during COVID-19”, *Nursing Ethics*, Vol 29 No. 6, pp. 1441–1456. | Unsuitable participants |
| Bedzow, I., Wynia, M.K. and Brendel, R.W. (2025). ”From Awareness to Action: teaching ethical decision-making to health-care leaders”, *Perspectives in Biology and Medicine,* Vol 68, No. 2, pp. 297-313. | Full text is not available |
| Blomqvist, H., Bergdahl, E., and Hemberg, J. (2022). “Ethical sensitivity and compassion in home care: Leaders’ views”, *Nursing Ethics, Vol* 30 No. 2, pp. 180–196. | Unsuitable phenomenon |
| Bogdasarian, R. (2020). “Leadership, ethics and the adoption of evidence-based medicine among leaders of internal medicine and surgery*”, Encyclopedia of Surgery,* Vol 1, pp. 6381–6391. | Unsuitable study design |
| Camillo, C. A. (2023). “Addressing the ethical problem of underdiagnosis in the post-pandemic Canadian healthcare system”, *Healthcare Management Forum*, Vol 36 No. 6, pp. 420–423. | Unsuitable phenomenon |
| Campbell, L. (2020). Organisation ethics, relational leadership and nursing. In H. Kohlen and J. McCarthy (Eds.), *Nursing ethics.* | Unsuitable study type |
| Chan, H. Y., Zhao, Y.-Y., Liu, L., Chong, Y.-Y., Cheng, H.-Y. and Chien, W. T. (2022). “Ethical challenges experienced by care home staff during COVID-19 pandemic”, *Nursing Ethics*, Vol 29 No. 7-8, pp. 1750–1760. | Unsuitable participants |
| Cikotić, S. (2021). Leadership in complex situations. In Ş. Ş. Erçetin, Ş. N. Açıkalın and E. Vajzović (Eds.), Chaos, complexity and leadership. | Full text is not available |
| Danis, M., Fox, E., Tarzian, A. and Christopher, C. D. (2021). “Health care ethics programs in U.S. Hospitals: results from a National Survey”, *BMC Med Ethics*, Vol 22 No. 107. | Unsuitable phenomenon |
| Frolic, A. and Miller, P. (2022). “Implementation of Medical Assistance in Dying as Organizational Ethics Challenge: A Method of Engagement for Building Trust, Keeping Peace and Transforming Practice”, *HEC Forum,* No. 34, pp. 371–390. | Unsuitable phenomenon |
| Flynn, G., Smith, D. and Kirwan, M. (2022). “Leadership, ethics, and law: A response to the coronavirus pandemic”, In G. Flynn (Ed.), *Leadership and business ethics*. | Unsuitable study type |
| Gassas, R., Ahmed, M. E., Asloob, M. (2024). “Ethical Decision-Making Confidence and Professional Values Among Nurse Leaders”, *The Journal of Nursing Administration,* Vol 54 No. 6, pp. 353-360. | Unsuitable phenomenon |
| González-García, A., Pinto-Carral, A., Marqués-Sánchez, P., Liebana-Presa, C., García-Fernández, R. and Pérez-González, S. (2025). “Characteristics of the Competency Ethical Principles for the Nurse Manager: A Systematic Review”, *Journal of Nursing Management.* | Unsuitable study design |
| Grubb, B. (2024). “Styles, strategies, and challenges of PA leaders nationally: An exploratory qualitative study”, *the Journal of the American Academy of Physician Associates,* Vol 37 No. 11, pp. 38-42. | Unsuitable phenomenon |
| Hansen, F.T. and Jørgensen, L B. (2021). “Wonder-inspired leadership: Cultivating ethical and phenomenon-led healthcare”, *Nursing Ethics*, Vol 28 No. 6, pp. 951-966. | Unsuitable phenomenon |
| Hasan, F., Ssahoo, A. K., Goyal, W., Seth, K., Jauakumar, S. S. and Sikchi, S. R. (2024). Leadership and Ethical Decision-Making in Healthcare Management. *Health leadership and quality of life*, 3. | Unsuitable phenomenon |
| Hashemi, E. and Barkhordari-Sharifabad, M. and Salaree, M. M. (2020). “Relationship between Ethical Leadership, Moral Distress and Turnover Intention from the Nurses’ Perspective!”, *Iranian journal of medical ethics and history of medifine,* Vol 13 No. 1, pp. 552-563. | Unsuitable language |
| Helm-Murtagh, S. and Erwin, P. (2022). “Leadership in practice: Essentials for healthcare and public health leaders”, *Leaders*, pp. 1-489. | Unsuitable study type |
| Ilse, R. (2024). “Ethical challenges in healthcare innovation: A leadership perspective”, *Healthcare Management Forum*, Vol 37 No. 6, pp. 467-470. | Unsuitable phenomenon |
| Jakobsen. L., Olsen, R.M., Brinchmann, B.S. and Devik, S.A. (2024).  ”Digital ethical reflection in home nursing care: Nurse leaders’ and nurses’ experiences”, Nursing Ethics, Vol 32 No. 1, pp. 186-200. | Unsuitable phenomenon |
| Jian, Q., Wang, X., Al-Smadi, H. M., Waheed, A., Badulescu, A. and Samad, S. (2022). “Proposing a Robust Model to Reduce Employees’ Turnover Intentions in an Ethical Leadership Framework: Empirical Evidence from the Healthcare Sector”, *International Journal of Environmental Research and Public Health,* Vol 19 No. 15, pp. 8939. | Unsuitable participants |
| Kemi, O., Adaora, I.O. (2023). "Responsible Leadership for the New Normal: Ensuring Fairness in Business and Health", *Responsible Management of Shifts in Work Modes – Values for Post Pandemic Sustainability*. Pandemic sustainability, No. 2, pp. 1-19. | Unsuitable study type |
| Keselman, D. and Saxe-Braithwaite, M. (2020). ”Authentic and ethical leadership during a crisis”, *Healthcare Management Forum,* Vol 34 No. 3, pp. 154-157. | Unsuitable phenomenon |
| Koskinen, C., Kaldestad, K., Rossavik, B. D., Jensen, A. R. and Bjerga, G. (2022). ”Leader as maintainer of multi-professional ethical competence in healthcare practice”, *Hoitotiede,* No. 34, pp. 53-61. | Unsuitable phenomenon |
| Ledandrini, J. (2025). “Culture: Addressing Workforce Challenges”, *Healthcare* *Executive,* Vol 40 No. 3, pp. 26-29. | Full text is not available |
| Liu, O., Grieb, S.M., Halsey, J.N., Levine, R.B., Oliva-Hemker, M., Lee, J.K. (2024). “Becoming Leaders – A Qualitative Research Study on the Priorities and Concerns of Early Career Women Faculty in Academic Medicine”, *Journal of* *Healthcare Leadership*, No. 16, pp. 511-523. | Unsuitable participants |
| Marcantonio, M. D. and Valentini, I. (2023). “Empirical research findings on ethics and leadership in the Italian healthcare system”, *Mecosan,* No. 126, pp. 29–46. | Unsuitable language |
| Martinelli, L.A. (2021*). Stakeholders and Ethics in Healthcare: Ethical Accountability for Organizations* (1st ed.). Routledge. | Unsuitable study type |
| Markey, K., Moloney, M., Doody, O. and Robinson, S. (2022). “Time to re-envisage integrity among nurse leaders”, *Journal of nursing management,* Vol 30 No. 7, pp. 2236-2240. | Unsuitable phenomenon |
| Majidipour, P., Hamtan, K. B., Marzooghi, R. and Salehi, M. (2021). ”Women’s educational supervisors’ experiences of leadership challenges due to care ethics in Kermanshah hospitals”, *Journal of Advanced Pharmacy Education and Research,* Vol 11 No. 4, pp. 121-126. | Full text is not available |
| McCruden, P.J. (2022*). The Tension Between “Margin and Mission” as an Ethical Issue in Healthcare.* In: Wasson, K., Kuczewski, M. (eds) Thorny Issues in Clinical Ethics Consultation. Philosophy and Medicine, 143. Springer, Cham. | Unsuitable study type |
| Menghwar, P.S., Homberg, F., Zaidi, Z. et al. (2025). “Rethinking Crisis as Expected: Stakeholder Leadership in Navigating Ethical Dilemmas and Avoiding Polycrises”, *Journal of business ethics.* | Unsuitable participants |
| Miller, P.H., Epstein, E.G., Smith, T.B., Welch, T.D., Smith, M., Bail, J.R. (2023). “Moral distress among nurse leaders: A qualitative systematic review”, *Nursing* *Ethics,* Vol 30 No. 7-8, pp. 939-959. | Unsuitable study design |
| Miller, K. and Hartsock, J. (2023). “Developing a Clinical Organizational Ethics Program”, *Journal of Hospital Ethics*, Vol 9 No. 1, pp. 12-19. | Full text is not available |
| Muhammad, A. F., Ishfaq, A., Insya, A. and Zanaira, I. (2021). ”Authentic leadership and follower’s role ethnicality: the role of leader’s ethical voice and ethical culture”, *International Journal of Ethics and Systems,* Vol 37 No. 3, pp. 422–441. | Unsuitable participants |
| Munezhi, M. and Hammad, N. (2020). “Ethical health leadership: Lessons from low- and middle-income countries during COVID-19”, *Healthcare Management* *Forum*, Vol 34 No. 1, pp. 62-67. | Unsuitable phenomenon |
| Munkeby, H., Moe, A., Bratberg, G. and Devik, S. A. (2022). “Ethics between the lines’ – nurses’ experiences of ethical challenges in long-term care”, *Global Qualitative Nursing Research*, 8, Article 23333936221089158. | Unsuitable participants |
| Mushtaq, A. and Muhammad, I. K. (2023). ”Beyond the universal perception: Unveiling the paradoxical impact of ethical leadership on employees’ unethical pro-organizational behavior”. *Heliyon*, Vol 9 No. 11, e20217. | Unsuitable participants |
| Musto, L., Schreiber, R. and Rodney, P. (2021). “Risking vulnerability: Enacting moral agency in the is/ought gap in mental health care”, *Journal of Advanced Nursing*, Vol 77 No. 5, pp. 2358-2471. | Unsuitable phenomenon |
| Ocho, O. N., Pieper, B., Pulcini, J. and Wheeler, E. (2020). “ET/WOC nursing -- leadership lessons learned from the COVID-19 pandemic: an opinion”, *World* *Council of enterostomal therapists journal,* Vol 40 No. 3, pp. 43-46. | Full text is not available |
| Patne, S. A. and Kanyal, D. (2024). ”Exploring ethical implications in hospital management: Aligning business objectives with patient care ethics”, *Muultidisciplinary reviews*, Vol 7 No. 10. | Unsuitable study design |
| Relias LLC. (2024). “Nursing leaders need ethicists' help with moral distress”, *Medical Ethics Advisor*, Vol 40 No. 5, pp. 1-16. | Unsuitable phenomenon |
| Rindlisbacher, D. (2020). “Coach Mindset: Preparing Leaders to Create a Climate of Trust and Value”, *Nursing Administration Quarterly*, Vol 44 No. 3, pp. 251-256. | Full text is not available |
| Saleem, M., Qadeer, F., Mahmood, F., Han, H., Giorgi, G. and Ariza-Montes, A. (2021). “Inculcation of Green Behavior in Employees: A Multilevel Moderated Mediation Approach*”, International Journal of Environmental Research and Public Health*, Vol 18 No. 1, pp. 331. | Unsuitable phenomenon |
| Sier, V.Q. (2025). “Future of medical leadership in the age of artificial intelligence”, *BMJ Leader.* | Full text is not available |
| Sowunmi, O. (2022). “The Prospect of Employees’ Job Satisfaction Under Ethical Leader-Member Exchange Relationship”, ProQuest dissertations and theses. | Unsuitable study type |
| Stephen, J. M. and Zoucha, R. (2021). “A Call for Nurse Leader Action: Ethical Nursing Care of Latinx Unauthorized Immigrant Children and Families”, *Nurse* *leader,* Vol 19 No. 4, pp. 395-400. | Unsuitable phenomenon |
| Wei, S., Sial, M. S., Zhou, W., Badulescu, A. and Badulescu, D. (2021). ”Improving the Environmental Footprint through Employees: A Case of Female Leaders from the Perspective of CSR”, *International journal of environmental research and public* *health,* Vol 18 No. 24, pp. 13082. | Unsuitable participants |

Source: Authors´ own work.

Supplementary File 4. Summary of data extracted from research articles.

| Author, publication year, country of origin, language | Study aims | Participants, context | Study design, data collection, data analysis, | Key findings |
| --- | --- | --- | --- | --- |
| Aitamaa et al., 2021,  Finland | To investigate the frequency and difficulty of the ethical problems nurse managers encounter in their work and to determine the background factors correlating with the problems. | Nurse managers in ward, middle, and strategic management (*n* = 214) in Finland. | Cross-sectional survey design.    Data were collected with questionnaires.  Data were analysed statistically using the SAS 9.3 software package. Descriptive statistics, such as frequencies, means, and standard deviations, were used to describe the variables. | Ethical problems were related to the nursing staff; nurses’ experiences of being heard and giving positive feedback to them, and the organisation; allocation of resources. The most difficult ethical problems were related to the organisation. The most difficult ethical problems concern downsizing personnel due to cost-cutting. The next most difficult problems were tacit approval of workplace bullying and damage to the quality of nursing due to a focus on cost-effectiveness. Also, the lack of support from organisational administration, planning operations on doctors’ terms without regard for nursing input, and the sufficiency of financial resources and resources for development work were among the most difficult problems. |
| Arjama, A. et al., 2025, Finland | The aim was to describe the ethical issues encountered by nurse managers (NM). | 23 NMs from seven randomly selected organisations. | Qualitative study.  Data were collected by using semi-structured focus group interviews.  Data was analysed using inductive content analysis. | The ethical issues encountered by NMs are multidimensional and have both external and internal causes. NMs often deal with ethical issues on their own.  Ethical issues faced by NMs are related to 1) residents’ rights to self-determination; Being present when the staff are providing daily care, to recognise ethically challenging situations, being aware of the decisions related to the care residents receive, familiarity with residents and families to ensure person-centered care. 2) ethical decision-making about staff and procedures; Prioritising responsibilities during daily work, treating staff equally but also meeting the individual needs of staff, maintaining ethical decision-making ability, even when instructions are incomplete or rapidly changing. Most NMs did not have enough time to discharge all their responsibilities. They were committed to their roles, but felt their workloads were unfair. They felt it was unfair that they had to balance their time between taking care of the staffs’ occupational well-being, and their own, and that their work balance was affected by continuously working overtime. NMs also felt it was hard to take days off and, if they did, tasks accumulated that further increased their sense of burden. Fulfilling ethical leadership despite the conflicting roles of NMs in an organisation. Receiving support in ethically challenging situations from a supervisor or senior managers affected how well they could act ethically. Secondary tasks and misaligned support services leave less time for dealing with ethically important matters. Obligations to the employer even against the NMs’ own conscience. They knew that safety of staff and residents could not be guaranteed in all situations, and this weighed them down. They also felt bad if they promised family members individual care for a resident, knowing it could not happen due to staffing shortages. Defending ethics in long-term care settings on the societal level. They highlighted the importance of self-compassion and were aware that they needed to maintain high standards despite poor resources. Managing poorly resourced services and maintaining high standards. Balancing between regulations and daily care needs. In their opinion, statistics and monitoring took an unreasonable amount of their working time but could not achieve the well-being of residents or staff. The NMs felt that the number of statistics and monitoring required had increased in recent years, but they did not know who benefited from the information. |
| Beil-Hildebrand et al., 2024,  Germany, Austria, Switzerland, United States | The aim was to develop an understanding of distressing ethical issues nurse leaders face in the USA and three German-speaking European countries. | A total of 316 nurse leaders from the USA (n = 91), and from three German-speaking countries in Europe (n = 225) including Austria (n = 27), Germany (n = 114) and Switzerland (n = 84) participated in the online survey. | Descriptive cross-sectional study    Data were collected anonymously through the Qualtrics platform.    Data were analysed using a thematic analysis of the qualitative data. | Six themes identified as causing all nurse leaders moral distress included 1) a lack of individual and organizational integrity; violations such as cheating and plagiarism, 2) hierarchical and interprofessional issues; poor interdisciplinary communication and hierarchy subjecting patients to non-beneficial treatments or care that do not address their most immediate needs, 3) lack of nursing professionalism; incompetence, misbehavior, dishonesty, lack of accountability, poor teamwork, horizontal violence, 4) patient care/patient safety concerns; problems with skill and grade mix, excessive workload, staff shortages, lack of support services and time constraints preventing thorough nursing care, the failure to apply evidence-based care into nursing practice, inconsistencies in patient care, lack of access to specialty care, lack of palliative care and lack of effective pain management, 5) finances negatively impacting care and 6) issues around social justice. |
| Cunha et al., 2023,  Brazil | To understand the ethical problems experienced by nurse managers in the context of the COVID-19 pandemic. | 19 managers participate in the study.    The setting was a university hospital in Belo Horizonte, Brazil. | Qualitative study.    Data were collected through individual interviews with a semi-structured script and document analysis and analyzed using Content Analysis. | Three thematic categories were identified: 1) Ethical problems arising from duplication and lack of information. Nurse managers experienced ethical problems in the work process related to lack of information about standards and routines, lack of flow of patients with the diagnosis of COVID-19 and duplicity of information. Lack of knowledge by some professionals about the technical  work  instructions  related  to  patients  with  COVID-19; the lack of patient flow between sectors, especially in situations where procedures are performed outside the sector of origin; failure in the communication  of  the  diagnosis  of  the  patient  with suspected COVID-19 between the medical and nursing staff, causing  exposure  of  health  professionals  and  other  patients. 2) Ethical problems related to nursing staffing; from absenteeism, presenteeism, refusal of reassignment by the nursing technicians, and misunderstanding between the professional who wants to be in the COVID-19 sector to earn additional unhealthy salary, and the professional who does not want to be there due to fear of illness and 3) Ethical problems with a patient suspected or diagnosed with COVID-19. In  relation  to  the  patient,  some  of  the  problems  originated  in  the  multidisciplinary  team,  going  beyond nursing. In this way, in the view of some participants, negligence in the care occurred as in the cases in which medical professionals shirked patient care. |
| Chipps et al., 2022,  United States | To explore the professional and personal experiences of US nurse managers during the COVID-19 pandemic. | 39 nurse managers from 5 health systems across the US participated in focus groups.    Hospitals were located in the East, Mid-Atlantic, Midwest, and West Coast regions; ranged in size from 125 to 1250 beds; and included academic medical centers, midsize community hospitals, and rural hospitals. | A qualitative research design.    Data were collected in focus group sessions.    Data were analyzed using a constant comparative method. | Nurse managers faced many new and complex challenges during the pandemic including large-scale organizational challenges and unit-level challenges. Moral and ethical challenges: ethical and moral challenges associated with leadership decisions and patient care management. Moral/ethical issues related to visitors policies. Nurse managers experienced ethical challenges as a result of strains in hospital's finances. They expressed frustration that some staff members received crisis pay and others did not. Many experienced distress in conveying information from senior leadership that they felt very conflicted about. |
| Devik et al., 2020,  Norway | To explore and understand how nurse managers perceive their role in supporting their staff in conducting ethically sound care in nursing homes and home nursing care. | Ten nurse managers with human resources responsibilities for healthcare workers in four nursing home wards and six home nursing care districts.    The study was carried out in nursing homes and home nursing care services in three municipalities in mid-Norway. | A qualitative approach.    Data were collected by semi-structured interviews.    Data were analysed using content analysis. | Three main categories were identified: 1) managers’ perception of the importance of the role; Ethical challenges faced by the staff. Independent of the healthcare setting, all of the respondents noted that their work environments were characterized by heavy workloads and shortages of time and personnel. They could see that their staff were struggling to keep up with duties and the quality standard of care to patients with increasingly advanced healthcare needs. The conflict between giving optimal care and the manageable reality was seen as ethically challenging, and the staff’s handling of ethical challenges, 2) managers’ experiences of exercising the role; managers’ experiences of ethical work dealt mainly with participation in the national ethics project. Despite the fact that the project had survived to a small extent, managers had clear perceptions of both the value of ethical work and what prevented its implementation. Difficulties in prioritizing time for ethical reflection emerged as a huge barrier for the leaders. and 3) managers’ opportunities to fulfil their role. All managers stated that they strived to be close to their employees and to foster open and positive relationships. They were also concerned that the staff should feel that there was a low threshold for contacting managers. Challenges with conceptualising ethics were highlighted, as well as a lack of applicable tools or time and varying motivation among employees. |
| Ghimire, A. et al., 2025, Nepal | The purpose of this study is to explore the lived experiences of Nepalese nurse managers as they grapple with these ethical dilemmas created by brain drain (The relentless exodus of skilled healthcare professionals from low- and middle-income countries to wealthier nations). | Ten nurse managers (five from an urban hospital and five from a rural hospital) in Nepal. | Qualitative descriptive study.  Semi-structured interviews and inductive content analysis. | Thematic analysis revealed five core themes: 1) Moral Distress on the Frontlines: They frequently find themselves in situations where they must balance their professional obligation to provide optimal patient care with the significant limitations caused by understaffing and a lack of resources. This statement underscores the agonizing choices nurse managers must make when resources are scarce, forcing them to compromise ideal care and live with the consequences. It also highlights the often invisible emotional labor involved in leading during a crisis, further contributing to their moral distress. 2) Unequal Burden, Unequal Access. This quote underscores the growing gap between healthcare needs and the availability of skilled professionals in rural areas, highlighting the sense of injustice felt by nurse managers who witness the consequences. This theme demonstrates that brain drain is not just about an inadequate workforce but a social justice issue that exacerbates existing inequalities and undermines the fundamental right to healthcare. 3) The Ripple Effects of Exodus: The departure of skilled nurses creates a domino effect, negatively affecting workload, patient safety, mentorship, and the overall sustainability of the workforce. The loss of experienced nurses also has long-term consequences for the development of future healthcare professionals. This statement underscores the challenges of attracting and keeping qualified nurses in the face of global competition, forcing difficult decisions that can compromise the quality of care. It also highlights the ethical dilemmas inherent in balancing staffing needs with the imperative to provide safe and effective care. 4) Beyond the Hippocratic Oath. The departure of each nurse is perceived as a dual phenomenon: it signifies a celebration of individual career advancement while simultaneously representing a collective loss within the healthcare team. 5) Policy at the Crossroads. This perspective shifts the focus from individual choices to the systemic issues that drive brain drain, emphasising the need for policies that uphold the rights and well-being of healthcare workers. The difficulties in recruiting and retaining nurses, especially in rural areas. |
| Karlsson et al., 2024,  Sweden | To explore ethical challenges in relation to person-centeredness during the COVID-19 pandemic, from the perspective of leaders in residential care facilities. | A total of 26 leaders, 12 registered nurses and 14 first-line managers working in public or private residential care facilities in seven different municipalities in southern and northern Sweden, including rural and urban areas, participated in the study. | A qualitative study.    Data were collected with semi-structured interviews.    Data were analysed using conventional content analysis. | The overarching ethical challenge was having to disregard the individual needs of the person in order to protect the group and society. This included (a) Protecting the group versus promoting the older person’s autonomy; having to go against residents’ right to experience meaningfulness and engagement. Simultaneously, they had to consider what was best for all residents living there. Having to apply visitor restrictions despite the residents’ need for social contact. Prohibiting relatives from visiting even though the residents were at the end of their lives. Being forced to isolate the residents to avoid the spread of infection. Maintaining distance despite the residents’ need for closeness and touch. Going against a resident’s will by performing a COVID test or being forced to isolate them. (b) Being forced to lead care based on uncertainty instead of evidence; struggling with an endless flow of information but still being left with no answers. Having to apply guidelines that go against one’s personal beliefs. Standing alone in making decisions and being held accountable. (c) Striving to provide dignified care but lacking opportunities; Being unable to provide care based on professional competence because of inadequate resources. Having to establish prioritization schemes for care, at the cost of individual needs. (d) Going far beyond ordinary duty and endangering one’s own and the staff’s health. Being compelled to protect the staff but still having to expose them to infection. Feeling compelled to prioritize work over personal life. Being forced to pressure staff to shoulder an unreasonable workload and risk crossing their limits. |
| Musbahi et al., 2022,  United Kingdom | To investigate the impact of COVID-19 on ethical leadership principles using a validated quantitative survey of National Health Service (NHS) leaders to compare pre- and post pandemic ethical leadership principles. | A total of 79 responses were received.    Inclusion criteria included consultants and registrars leading clinical teams or NHS managers, senior nurses, and matrons. | A quantitative survey.    Data were collected with a questionnaire.    Data were analysed statistically using SPSS, and Chi-squared testing was performed. | Leaders were more likely post-pandemic to speak out against injustice and unfair practices. Leaders were also more concerned with matters of human dignity as well as understanding how some groups may be privileged. |
| Nopita Wati et al., 2023,  Indonesia | To explore nurse team leaders’ experiences regarding strategies and challenges in dealing with ethical problems in hospital settings in Indonesia | 14 nurse team leaders  were included.    The respondents were selected from seven hospitals (three public and four private hospitals) in Indonesia. | A qualitative study employed a hermeneutic phenomenology design.    Data were collected with semi-structured interviews.  Data were analysed by Van Manen’s approach. | The challenges for the nurse team leaders in resolving ethical problems consisted of (i) seniority. Seniors have a big responsibility as models, and juniors bring new hope because they are up to date with the latest knowledge. (ii) Trust issues: dealing with ethical challenges, nurses must have excellent teamwork, which requires trust. and (iii) lack of reflection and ethics training. The participants stated that past mistakes made by the nurses should be discussed in pre- or post-conferences during shifts or in a forum to avoid repeating them in the future. This should also be supported by the hospital to provide nurses with ethics training to raise awareness. |
| Storaker et al., 2022,  Norway | To gain deeper insight into how nurses as leaders in somatic hospitals describe ethical challenges. | Nine female nurses and one male nurse. Nine were nurse leaders on clinical wards, while one was head of an outpatient facility. | A qualitative study.    Data were collected through interviews.    Data were analysed using a hermeneutical approach. | Four main areas were identified: 1) deficient ethical language. Most of the participants did not use ethical terms or ethical language. Thus, it may be difficult to interpret exactly how they understood it. All of them talked about situations where ethical issues were obvious, but they did not address them as ethical situations. The example above confirms that this leader was concerned about the patient’s vulnerability. 2) conflicting demands on nurse leaders regarding staff management. They were concerned about communication in general and underlined the importance of talking in a caring way to patients and of talking respectfully and openly to other members of staff. All the participants reported that personnel matters represented their most challenging task as nurse leaders. They had to conduct employee interviews with everyone once a year, and they demanded of themselves that each employee should be treated fairly. Justice and equality appeared to be values to which the participants gave high priority. They spent time and energy on being fair. However, the principle of fairness seemed to be associated with working conditions such as shift rota. 3) concerns regarding young nurses’ ethical consciousness. The nurse leaders expressed concerns about changes they were seeing among the young nurses compared to previous years, and that they seemed immature and less reflective. They seemed to consider their job as an opportunity to make new friends and have fun. Moreover, they no longer seemed to subscribe to the idea of nursing as a calling. In sum, these findings confirm the nurse leaders’ ability and willingness to guide young nurses in giving high-quality patient care. and 4) restricting factors on the creation of a climate of ethics. The participants described their work and duties as a constant squeeze in different directions. The experience of being pulled in different directions at the same time, such as budget meetings, challenging patient situations, and vacant shifts, brought them little peace of mind. As far as complex patient needs and medical treatment are concerned, there is an increasing demand for high levels of nursing competence. Procedures that used to be performed by physicians, such as cytostatic infusions, were now being performed by nurses. In addition, senior management seemed more concerned with keeping within budgets than with creating an ethical climate. |

Source: Authors´ own work.

Supplementary File 5. Summary of data extracted from anecdotal articles.

| Author, publication year, country of origin, language | Type of publication, focus of paper | Focal groups, concepts, context | Key relevant points |
| --- | --- | --- | --- |
| Arcadi, P., 2025, Italy | Theoretical-reflective paper.  Explores the ethical dimensions of AI from a nursing perspective, focusing on the role of nurse leaders as ethical agents in the digital transformation of care. | Nurse leaders  Artificial intelligence (AI)  Ethical dimensions  Healthcare | The integration of artificial intelligence (AI) into healthcare marks a paradigmatic shift, raising not only technical and organisational challenges but also profound ethical questions. Nurse leaders are called to act along three ethical pillars: 1) fostering transparency in algorithmic processes; Nursing leaders are called to act as mediators of technological meaning, ensuring that digital processes are translated into understandable, ethically sound, and clinically relevant information, 2) ensuring shared responsibility for AI-supported decisions; Nursing leadership must actively engage in identifying and mitigating algorithmic harms, ensuring that AI applications do not perpetuate disparities but instead promote equity, inclusion, and justice. This involves promoting inclusive data practices, demanding transparency from developers, and embedding ethical reflection into care practices and organisational decisions, and 3) sustaining human relationships as the core of care; nursing leadership must uphold and cultivate an organisational culture where person-to-person relationships remain central. By reclaiming time for presence and promoting emotionally intelligent leadership, nurses can foster collaborative environments that support the well-being of both patients and healthcare teams.  Nurse leaders must act as ethical agents capable of humanising technology, preventing depersonalization, and safeguarding the fundamental values of care. They play a key role in ensuring that innovation aligns with justice, well-being, sustainability, and the dignity of each individual.  Facial recognition technologies have shown higher error rates when identifying people of color compared to White individuals, and studies have revealed that algorithms used to predict healthcare needs often underestimate the severity of illness in Black patients compared to White patients with the same algorithmic risk score. These biases can lead to unequal treatment and reduced access to appropriate care.  The growing interaction between humans and machines may further weaken interpersonal relationships, which are already fragile in a society marked by social isolation and technological mediation. Especially among younger generations, the digitalisation of communication risks reducing opportunities for authentic human connection. In this scenario, safeguarding and promoting relational time becomes not only a professional priority but also a societal and ethical imperative. |
| Downs et al., 2022,  United States | Paper    This paper discusses 3 ethical challenges: workforce shortages, staff assignments, and moral distress. It also offers strategies to leverage our code of ethics to navigate these situations. | Nurse leaders  Ethical challenges  Healthcare | The COVID-19 pandemic has presented numerous ethical challenges for nurse leaders.  Many nurse leaders today are feeling overwhelmed. They are attempting to promote and sustain ethics while facing incredible clinical challenges and supporting a workforce that is fragmented, understaffed, and burned out. Many nurse leaders can chronicle multiple ethical challenges that have emerged while making staffing assignments related to COVID-19 patients. The struggle and toil of being fair to our team and exposing them to the least physical and emotional harm possible are real. Nurse leaders are struggling to balance the well-being of the patients and team, all the while recognising that staff’s well-being directly impacts the care they provide. Staffing and workforce scarcity is one of the most pressing challenges facing our nurse leaders today. The workforce scarcity poses an ethical challenge for nurse leaders trying to determine whether the quality of care is being sacrificed. Nurse leaders are encouraged to utilise the clinical ethics resources available to help prevent and mitigate moral distress. They must feel empowered to call on ethics experts to help gain the expertise they need to lead through these morally distressing situations. Even nurse leaders who are untrained in ethics and have little access to ethics resources can mitigate moral distress and promote resiliency within their teams. |
| Ennis-O-Connor and Mannion, 2020,  Ireland | Paper    This article describes the changing parameters of professional conduct in digital environments and proposes a set of considerations and recommendations for health leaders to navigate this new frontier. | Healthcare leaders  Social media challenges  Healthcare | How health leaders address issues of social media usage in an organisation has an impact on public confidence and engagement. Health leaders must support employees to meet their ethical obligations in the digital age. Organisations should not only provide policies and guidelines but also educate and support employees on appropriate social media use. Organisations must offer practical strategies to maximise the benefits of social media while respecting ethical considerations. Healthcare leaders will need to apply principles of professionalism in new settings to establish guidelines and policies that promote ethical conduct. |
| Lahey et al., 2021,  United States | Feature    To discuss how organizational ethics has supported senior leaders in two tertiary hospitals during institutional responses to the COVID-19 pandemic. | A team of ethicists and senior leaders of two New England tertiary care centers.  Challenging decisions in Healthcare | Leaders make challenging decisions during the pandemic, from fair ventilator allocation and PPE equity to visitation policies, protecting resuscitation team safety in the event of PPE shortages, managing patient refusal of masks and testing, addressing staff moral distress, and overseeing learners. |
| Lesandrini and Leclerc, 2024,  United States | Paper    This paper examines the importance of ethical leadership in health care, why formal training beyond academic preparation is vital, and how leaders can advocate for ongoing support and development. | Nurse leaders  Ethical leadership Challenges faced by nurse leaders  Healthcare | Nurse leaders face tremendous challenges, including stressful workloads, staffing issues, safety concerns, consumerism, financial constraints, and rapidly evolving technology, all with ethical undertones. Nurses are considered leaders in every role, from the bedside to the boardroom. As such, nurses are advocates for protecting patient rights, dignity, and autonomy, and they are role models for colleagues by living and leading with ethical behaviors. |
| Lesandrini and Reis, 2022,  United States | A case study scenario    The case study scenarios presented here demonstrate examples of common ethical staffing challenges that healthcare leaders have faced, notably the allocation of care providers among COVID-19 patients and the balancing of care quality with staff and patient safety. | Healthcare leaders  Ethical staffing challenges  Healthcare | The ethical challenges that have arisen include those related to resource allocation, visitation policies, and the appropriateness of do-not-resuscitate orders.  Besides workforce shortages, there are other ethical challenges related to staffing. For example, new staffing models such as telehealth, broader integration of students, and the expanding scope of advanced practice providers appear, at face value, to be beneficial to patients, communities, and health systems. Nevertheless, healthcare leaders who adopt these models should first thoroughly consider the ethical implications of such decisions for all involved. They must identify the values that are promoted and demoted by each potential choice.  They struggled over the principle of fairness in this situation. The staffing case study scenarios presented here also show how ethical challenges in healthcare can be more complex than a single issue and can present many competing values. This is especially true in situations of resource allocation, such as those related to the workforce. Thus, leaders must not only recognise the ethical considerations of an issue but also learn to balance them. They must be able to ethically distribute insufficient resources. |
| Rushton, 2025,  United States | Theoretical paper  An overview of ethical challenges nurse leaders face and integrity preserving practices to reduce the detrimental effects of ethical challenges and moral residue. | Nurse leaders  Ethical challenges  Healthcare | Many nurse managers have an unreasonable scope of responsibility that includes a disproportionate and unrealistic number of direct reports. They may be held accountable for patient, organisational, or nurse retention outcomes that are multifactorial and largely beyond their control.    Given their current staff shortage, she is also concerned about the impact of this decision on the quality of patient care and nurse morale. In ethical terms, Wanda is worried about respect for patients and nurses, achieving a reasonable balance of benefits and burdens for everyone, and ensuring fair and equitable resource allocation.    Nurse leaders may lack clarity about their core values, they may be confused when there is incomplete or dissonant information or they may struggle to reconcile the ethical trade-offs that accompany complex decisions made by others that they are expected to implement. |
| Rushton et al., 2025, United States | This article presents a multifaceted ethical decision-making framework that helps identify risks, evaluate ethical tradeoffs, and guide decisions, offering tools for more intentional decision-making and communication. | Nurse leaders  Ethical framework  Healthcare | Nursing leaders must balance competing priorities and ethical considerations when making workforce decisions. Nurse leaders must navigate the delicate balance between ensuring safe, high-quality patient care; fostering inclusive stakeholder engagement; and maintaining a healthy, supportive environment, all while addressing organisational and financial priorities, managing limited resources, and responding to external pressures that can distract from the nursing score mission. Ethical decision-making for Chief Nursing Officers is both challenging and necessary when balancing the demands of patient experience, workforce engagement and sustainability, innovation, and the organisation's bottom line, especially as new care models are introduced. When making decisions about potential workforce strategies and care models, senior nursing leaders are frequently tasked with balancing multiple competing priorities and considerations |
| Stucky and Wymer, 2023,  Germany, United States | Theoretical paper    The purpose of this manuscript is to educate nurse leaders on the critical adaptive competency of ethical reasoning to guide just and strategic ethical decision-making for their organisations and communities. | Nurse leaders  Challenges faced by nurse leaders  Healthcare | Nurse leaders face multiple challenges, including workforce shortages, financial concerns, changing technologies, government mandates, and issues impacting patient safety and healthcare quality. The COVID-19 pandemic and humanitarian missions drove nurse leaders to make critical, life-saving decisions amid uncertain processes, an ever-changing operational picture, incomplete data, and conflicting organisational and governmental directives. |
| Sullivan et al., 2024,  United States | Paper    This article explores the historical development and recent surge of artificial intelligence (AI) technologies in healthcare, focusing on the role of the future nurse leader. | Nurse leaders  Artificial intelligence Healthcare | Nursing leadership is witnessing a profound transformation, with artificial Intelligence (AI) playing a pivotal role in reshaping how healthcare systems are managed and patient care is delivered.  However, some challenges must be considered, such as ethical and legal concerns, and skill training will be needed for nurses and nurse leaders.    Challenges incorporating AI technology into nursing brings forth challenges encompassing legal, regulatory, and ethical dimensions. Leaders grapple with decisions regarding data accessibility amidst privacy concerns, the varied nature of electronic health records, and the need to address biases in data management. Furthermore, leaders must acknowledge and address nurses' concerns about the potential erosion of human interaction to foster trust in the utilisation of AI-generated data.  Nurse leaders must stay up to date on emerging technologies in patient care to optimise resource allocation, manage costs effectively, and plan strategically, all while actively participating in the governance of these technologies. |

Source: Authors´ own work.
